# Regulatory Mechanisms of a Highly Pectinolytic Mutant of *Penicillium occitanis* and Functional Analysis of a Candidate Gene in the Plant Pathogen *Fusarium oxysporum*

**DOI:** 10.3389/fmicb.2017.01627

**Published:** 2017-09-08

**Authors:** Gustavo Bravo-Ruiz, Azza Hadj Sassi, Marina Marcet-Houben, Antonio Di Pietro, Ali Gargouri, Toni Gabaldon, M. Isabel G. Roncero

**Affiliations:** ^1^Departamento de Genetica, Universidad de Cordoba and Campus de Excelencia Agroalimentario (ceiA3) Cordoba, Spain; ^2^Bioinformatics and Genomics Programme, Centre for Genomic Regulation, The Barcelona Institute of Science and Technology Barcelona, Spain; ^3^Universitat Pompeu Fabra Barcelona, Spain; ^4^Laboratoire de Biotechnologie Moléculaire des Eucaryotes, Centre de Biotechnologie de Sfax Sfax, Tunisia; ^5^Institucio Catalana de Recerca i Estudis Avançats Barcelona, Spain

**Keywords:** pectinases, cellulases, complex polysaccharide degradation, Clb-like transcription factor, gene regulation

## Abstract

*Penicillium occitanis* is a model system for enzymatic regulation. A mutant strain exhibiting constitutive overproduction of different pectinolytic enzymes both under inducing (pectin) or repressing conditions (glucose) was previously isolated after chemical mutagenesis. In order to identify the molecular basis of this regulatory mechanism, the genomes of the wild type and the derived mutant strain were sequenced and compared, providing the first reference genome for this species. We used a phylogenomic approach to compare *P. occitanis* with other pectinolytic fungi and to trace expansions of gene families involved in carbohydrate degradation. Genome comparison between wild type and mutant identified seven mutations associated with predicted proteins. The most likely candidate was a mutation in a highly conserved serine residue of a conserved fungal protein containing a GAL4-like Zn_2_Cys_6_ binuclear cluster DNA-binding domain and a fungus-specific transcription factor regulatory middle homology region. To functionally characterize the role of this candidate gene, the mutation was recapitulated in the predicted orthologue *Fusarium oxysporum*, a vascular wilt pathogen which secretes a wide array of plant cell wall degrading enzymes, including polygalacturonases, pectate lyases, xylanases and proteases, all of which contribute to infection. However, neither the null mutant nor a mutant carrying the analogous point mutation exhibited a deregulation of pectinolytic enzymes. The availability, annotation and phylogenomic analysis of the *P. occitanis* genome sequence represents an important resource for understanding the evolution and biology of this species, and sets the basis for the discovery of new genes of biotechnological interest for the degradation of complex polysaccharides.

## Introduction

Pectins are complex heteropolysaccharides that are present mainly in the middle lamellae and primary cell walls of higher plants. Four substructures of pectin have been identified. The most abundant one is polygalacturonic acid (PGA) which consists of α backbone of D-galacturonic acid (GA) residues linked by α-1,4-glycosydic bonds (Hoondal et al., 2002; Mohnen, 2008). Pectinases encompass a family of enzymes able to degrade the pectin polymer. Microbial pectinolytic activity derives from the secretion of different classes of pectinases with distinct modes of action on pectin and PGA. For example, endo-polygalacturonases (endoPGs) specifically cleave the α-1,4 -glycosydic bond between two non-methylated GA residues.

The production of microbial pectinases is often subject to substrate induction and carbon catabolite repression (De Vries et al., 2002). Coordinated induction of genes encoding extracellular enzymes and sugar uptake systems in fungi is often mediated by Zn2Cys6 TFs that bind to conserved promoter elements in the co-regulated genes (Tani et al., 2014). With few exceptions (Alazi et al., 2016), the mechanisms underpinning transcriptional regulation of pectinase genes remain unknown in most fungal species.

*Fusarium oxysporum* is a devastating fungal pathogen that secretes a wide array of plant cell wall degrading enzymes (CWDEs), including polygalacturonases (Bravo-Ruiz et al., 2016), lipases (Bravo-Ruiz et al., 2013), pectate lyases (Huertas-González et al., 1999), xylanases (Gómez-Gómez et al., 2001, 2002), and proteases (Di Pietro et al., 2001), all of which contribute to plant infection. Many of these functions are encoded by redundant genes, which difficults the characterization of their roles by targeted gene deletion. One way to overcome the limitation of functional redundancy is to inactivate the transcription factors that regulate the coordinate induction or repression of multiple genes.

The pectinolytic system of *Penicillium occitanis* represents a model system for enzymatic regulation that has been intensely studied (Hadj-Taieb et al., 2002; Trigui-Lahiani and Gargouri, 2007). A mutant exhibiting constitutive and specific overproduction of pectinases was previously isolated by classical nitrous acid mutagenesis. The mutant secretes large amounts of different pectinolytic enzymes both when grown under inducing (pectin) or repressing conditions (glucose) (Trigui-Lahiani et al., 2008). The nature of the mutation(s) causing the pectinolytic overproduction phenotype is unknown.

In the present study we set out to identify the regulatory mechanism responsible for the pectinolytic overproduction phenotype of the CT1 mutant. To this aim we report, for the first time, the genomic organization of *P. occitanis* and compare the complete genome sequence of the parental and mutant strain. We further identified a putative Cys_6_Zn_2_ transcription factor which carries a mutation in a conserved residue in the CT1 mutant as a possible candidate for pectinolytic enzyme deregulation, and functionally tested this hypothesis by recapitulating the mutation in the orthologous gene of *F. oxysporum*.

## Materials and methods

### Fungal isolates and culture conditions

We note that given the closer relatedness of *P. occitanis* with *Talaromyces* species than to other *Penicillium* species, the denomination is phylogenetically inconsistent and will probably be subject to revision (Houbraken et al., 2014). However, given the current absence of an accepted alternative name, and the widespread use of *P. occitanis* in the literature, we will keep the *P. occitanis* denomination throughout the manuscript. The CT1 mutant was selected after a single round of nitrous acid (HNO_2_) mutagenesis from the wild type strain CL100 of *P. occitanis* (Hadj-Taieb et al., 2002) that was originally provided by G. Thiraby, Toulouse, France. Strains CL100 and CT1 are deposited at the culture collection of the Centre de Biotechnologie de Sfax, Tunisia, under reference numbers CTM10246 and CTM10496, respectively. The CT1 strain was routinely propagated on potato dextrose agar and maintained as spores in 20% glycerol at −80°C. *F. oxysporum* f.sp. *lycopersici* strain 4287 (race 2) was reported before (5). Microconidial suspensions were stored at −80°C with 30% glycerol. For extraction of DNA and microconidia production, cultures were grown in potato dextrose broth (PDB) (Difco, BD, USA) at 28°C as described (5). For induction conditions 5 × 10^8^ *Fusarium* microconidia were grown in PDB media for 14 h. The germlings were collected by filtration and transferred to flasks containing synthetic medium (SM) (Benoit et al., 2012) supplemented with one of the following carbon sources: 0.5% (w/v) pectin from citrus fruit (Sigma-Aldrich, Germany), 0.5% (w/v) glucose, 0.5% (w/v) pectin plus 0.5% (w/v) glucose, or 2.5% of tomato vascular tissue (TVT), as indicated, and cultures were maintained in a rotary shaker at 150 rpm and 28°C. Vegetative growth of mutants was determined on solid SM containing one of the following carbon sources: 1% (w/v) sucrose, 1% (w/v) cellobiose, 1% (w/v) insoluble cellulose, 1% (w/v) carboxymethylcellulose (CMC), 1% CMC+1% glucose, aliquots of 5 μL containing serial dilutions of freshly obtained microconidia (10^2^, 10^3^, or 10^4^) were spotted onto the agar plates and incubated at 28°C for 4 days before being scanned. Dry weight accumulation of mutants and wild type strain were determined in media containing complex (CMC, cellulose, or cellobiose) and simple carbon source (sucrose) after 120 h incubation at 28°C and 170 rpm.

### Genome sequencing and assembly

The two strains of *P. occitanis* (CL100 and CT1) were sequenced at the sequencing core facility of the Centre for Genomic Regulation using Illumina HiSeq 2000 technology and 50 bp-long single-end sequencing approach. Roughly 70 million reads were obtained for each strain providing a coverage between 91X and 102X.

Reads were filtered using a quality threshold of 10 and a minimum read length of 31. Genomes were then assembled using SPAdes (Bankevich et al., 2012) (see Supplementary Table 1 for statistics on the genome assembly). Gene prediction was performed using Augustus v2.0 (Keller et al., 2011) with parameters inferred for *A. nidulans* as used before in the annotation of *Penicillium* genomes (Ballester et al., 2015; Banani et al., 2016). The final annotation comprised 11,269 protein-coding genes. This Whole Genome Shotgun project has been deposited at DDBJ/ENA/GenBank under the accession NPFJ00000000 (*P. occitanis* CT1), and NPFK00000000 (*P. occitanis* CL100). The version described in this paper is version NPFJ01000000.

### Phylome reconstruction

A phylome, meaning the complete collection of phylogenetic trees for each gene encoded in a genome, was reconstructed for *P. occitanis* CL100. The pipeline used was previously described in Huerta-Cepas et al. (2004). Briefly the pipeline mimics the steps taken to reconstruct a phylogenetic tree while trying to optimize for accuracy and time. For each gene encoded in a genome (seed genome) a blast search is performed against a proteome database. In this case the proteome database was built using 29 species (Supplementary Table 4). Blast results were then filtered (*e* < 1e^−05^, overlap > 0.5) and the closest 150 homologs were aligned. The multiple sequence alignment was reconstructed using three different alignment programs MUSCLE v 3.8 (Edgar, 2004), MAFFT v6.712b (Katoh et al., 2005) and kalign (Lassmann and Sonnhammer, 2005), and each of the algorithms was used to align the proteins in forward and reverse orientation (Landan and Graur, 2007). A consensus alignment was obtained using M-coffee (Wallace et al., 2006) and then trimmed using trimAl v1.4 (Capella-Gutiérrez et al., 2009) (consistency-score cut-off 0.1667, gap-score cut-off 0.1). This final alignment was then used to reconstruct phylogenetic trees. Each tree was first reconstructed using a NJ approach as implemented in BIONJ (Gascuel, 1997), the likelihood of the topology was assessed using seven different models (JTT, LG, WAG, Blosum62, MtREV, VT, and Dayhoff). The model best fitting the data was then used to reconstruct a ML tree using phyML v3.0 (Guindon et al., 2010). Four rate categories were used and invariant positions were inferred from the data. Branch support was computed using an aLRT (approximate likelihood ratio test) based on a chi-square distribution. The best tree according to the AIC criteria was then chosen. The phylome, recorded with phylomeID 369 finally was formed by 11,004 trees. All the information is stored in phylomeDB v4 (http://phylomedb.org) (Huerta-Cepas et al., 2014).

### Phylome analysis

The phylome was scanned for the presence of species-specific expansions in *P. occitanis*. For each tree reconstructed in the phylome, ETE v3.0 (Huerta-Cepas et al., 2016) was used to search for monophyletic nodes that contained more than two sequences belonging to *P. occitanis*. The phylome was also scanned in order to identify orphan genes, understood as those genes that had no homologs in any of the species included in the phylome. 98 orphan genes were found that were present in the two *P. occitanis* strains. Orthologs and paralogs among the species considered were predicted using the species-overlap algorithm (Huerta-Cepas et al., 2007).

### Species tree reconstruction

**Figure 1:**
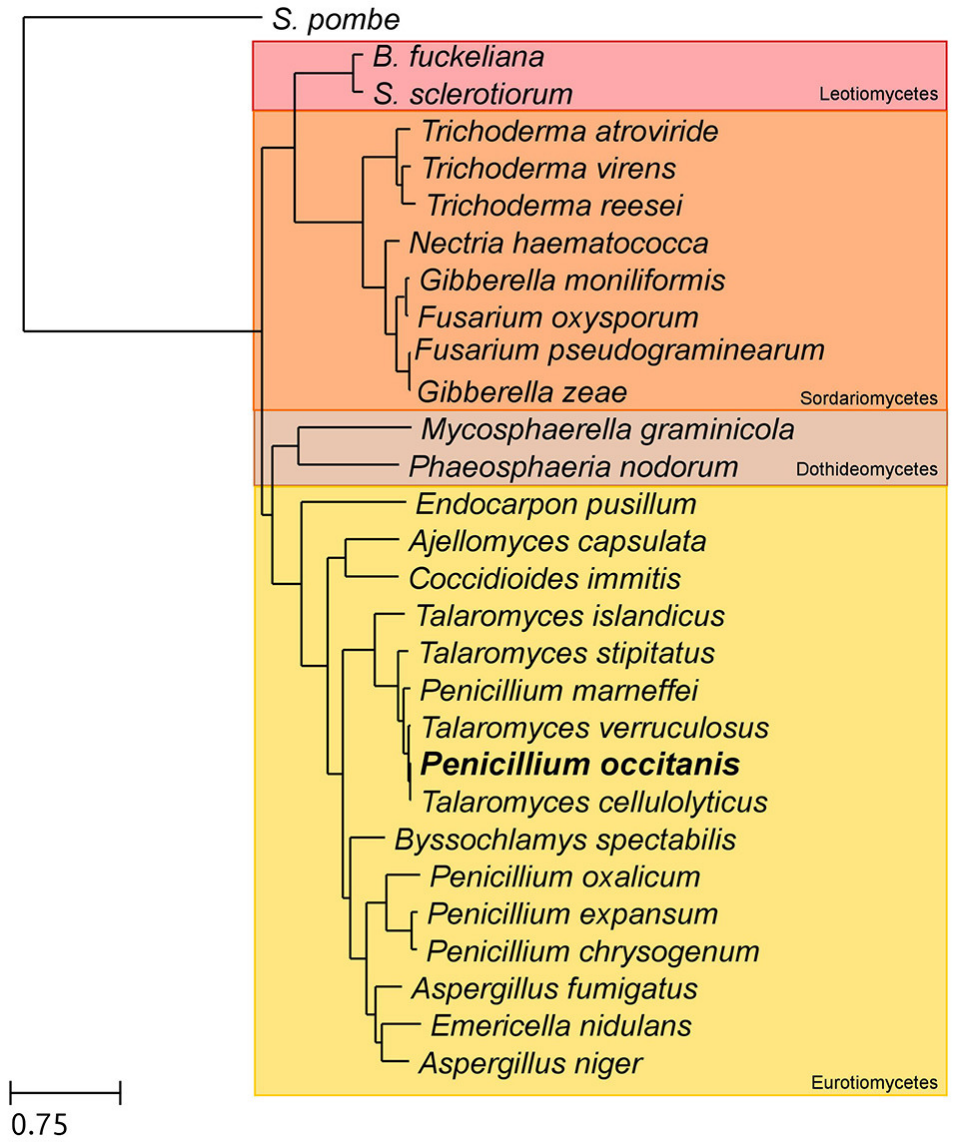
Species tree depicting the evolutionary relationships between the species included in the phylome. The tree is based on a concatenated alignment of 505 genes found in single copy in all 29 species included in the tree. In bold is the newly sequenced species *P. occitanis*.

A species tree was reconstructed using data obtained in the phylome of *P. occitanis*. 505 one-to-one orthologs that had only one copy in each of the 29 species used in the phylome were selected. The trimmed alignments of these proteins were then concatenated to form a single multiple sequence alignment, which was then used to reconstruct the species tree. The concatenated alignment had 3,77,573 amino acid positions. Fasttree (Price et al., 2010) was used to reconstruct the species tree using default parameters (Figure 1).

### Gene tree reconstruction

A broader tree of the transcription factor was reconstructed. We used the transcription factor found in *P. occitanis* and performed a blast search against uniprot, we limited our search to fungi. We selected the best 250 hits and filtered out strains and proteins that did not belong to a completely sequenced genome so as to limit the size of the resulting tree. The sequence of the identified *P. occitanis* Cys_6_Zn_2_ transcription factor was then added to the list and the tree was reconstructed by following the same pipeline used in the phylome reconstruction. ETE v3.0 was used to detect duplication and speciation nodes using a species overlap algorithm that assumes that a node is a duplication node if there are shared species at either side of the node, if this is not the case, the node will be a speciation node (Huerta-Cepas et al., 2007). The tree was rooted at the *Leotiomycetes* group.

### Comparison of *P. occitanis* strains

Reads of the two strains of *P. occitanis* were mapped to the assembly of the opposite strain using bwa v0.7.3a (Li and Durbin, 2009) aligning sequence reads, clone sequences and assembly contigs with BWA-MEM. SAMtools (Li et al., 2009) was then used to transform the sam files into bam files. GATK (McKenna et al., 2010) was then used to predict and filter SNPs. SNPs were then characterized depending on whether they were found in non-coding regions, promotor regions or coding regions. SNPs predictions were also compared between the two strains and a list of reciprocal SNPs was compiled (Table 1 and Supplementary Tables 5, 6).

**Table 1:** List of proteins that contained a reciprocal mutation found in the two *P. occitanis* strains.

**Protein PENO1**	**Protein PENOC**	**Function**
PENO1_019200	PENOC_048850	Ribonuclease CAF1
PENO1_011980	PENOC_010810	Hypothetical protein
PENO1_031020	PENOC_073480	Peptidase C48
PENO1_108040	PENOC_095050	Pre-rRNA-processing protein TSR2
PENO1_091170	PENOC_088330 (298V→298E)	Nucleic acid-binding, OB-fold
PENO1_027960	PENOC_037760 (198C→198S)	Transcription factor
PENO1_004030	PENOC_004030	alpha-ketoglutarate-dependent taurine dioxygenase

*First column indicates the code of the protein involved in the reference strain, the second column indicates the protein affected in the mutant strain and the third column indicates putative function*.

### Nucleic acid manipulation

Total RNA and genomic DNA were extracted from mycelium according to previously reported protocols (Raeder and Broda, 1985; Chomczynski and Sacchi, 1987). Southern analyses and probe labeling were carried out as described previously (Di Pietro and Roncero, 1998) using the non-isotopic digoxigenin labeling kit (Roche Life Sciences, Barcelona, Spain). Sequencing of DNA clones used in fungal transformation experiments was performed at STAB-VIDA (Setubal, Portugal). DNA and protein sequence databases were searched using the BLAST algorithm (Altschul et al., 1990) at the National Center for Biotechnology Information (NCBI) (Bethesda, MD) or at Broad Institute *Fusarium* comparative website http://www.broadinstitute.org. PCR reactions were routinely performed with the High Fidelity Template PCR system (Roche Life Sciences, Barcelona, Spain) or the Biotaq DNA polymerase (Bioline, Taunton, MA, USA).

### Transformation-mediated gene replacement and analysis of transformants

Targeted gene replacement of the FOXG_08883 *gene* was performed using the split marker technique (Catlett et al., 2003) as reported (López-Berges et al., 2010). The two overlapping DNA fragments were used to transform protoplasts of *F. oxysporum* wild type strain 4287, as reported previously (Di Pietro and Roncero, 1998). The resulting hygromycin resistant transformants were initially identified by PCR, and the homologous recombination event was confirmed by Southern analysis of selected transformants using an internal gene fragment as probe. Site-directed mutant allele FOXG_08883^160Ser>Cys^ was obtained by replacing the cytosine at position 599 of the sequence by guanine and the adenine 600 by thymine, using PCR amplification with a primer pair carrying these mutations (Table 2). This construct was used for co-transformation of protoplasts of the ΔFOXG_08883 mutant together with the phleomycin resistance cassette (Phl^R^) as selective marker (Punt and van den Hondel, 1992). The resulting phleomycin resistant transformants were initially identified by PCR and Southern analyses, and the presence of the mutated allele was confirmed by DNA sequencing.

**Table 2:** Oligonucleotides used in this study.

**Name**	**Sequence 5′–3′**	**Position to ATG**	**Experimental use**
**FOXG_08883**			
FOXG_08883-2	CTTTCAAGCGGGCGTGTCATA	−1474 (s)	Site-directed mutagenesis
FOXG_08883-4	GATGCTCGGGGTGTTATCTCT	+2696 (s)	Split marker
FOXG_08883-5	GCTGGGGGTCCTTCTCAACA	+3902 (as)	Site-directed mutagenesis/split marker
FOXG_08883-6	CCAGCCCTATACGACGGTCA	+3925 (as)	Site-directed mutagenesis/split marker
FOXG_08883-7	CCTGTCGCCAGGCAAACATG	+700 (s)	Site-directed mutagenesis
FOXG_08883-8	CATGTTTGCCTGGCGACAGG	+700 (as)	Site-directed mutagenesis
FOXG_08883-9	ggtacacttgtttagaggtaatcaactgAACTTACAGGTACAGAAC	+2 (as)	Split marker
FOXG_08883-11	ACTGTCGTGGCGGCAGGGA	−779 (s)	Split marker
FOXG_08883-12	GGCGTCGTTGATCGGGAGG	−691 (s)	Split marker
FOXG_08883-13	GGAGAGTTGAGAAGGATTTTGC	−430 (s)	Site-directed mutagenesis
FOXG_08883-14	ATGTTCTGTACCTGTAAGTTCAG	+1 (s)	Southern / mutants sequencing
FOXG_08883-16	CGATGCGACTGCTGCCAAAG	+1037 (as)	Southern / mutants sequencing
FOXG_08883-17	CCTTGTAACCAATGCGAGTCA	+183 (s)	RT-qPCR
FOXG_08883-18	ACCGATAATGAAGCCAGGTCT	+410 (as)	RT-qPCR
**PG**			
pg1-1	GTCACTTCGGGTACAAACATC	+790 (s)	RT-qPCR
pg1-2	CCTTGATGAACTTGATGCCGC	+1169 (as)	RT-qPCR
pgx4-1	GTACAGCATTGCCTCGCCAC	+275 (s)	RT-qPCR
pgx4-2	CGGGTTTCTCATTCGCAGGTT	+662 (as)	RT-qPCR
pg5-1	GCCTGGTCGCCTCCGTACT	+28 (s)	RT-qPCR
pg5-2	TCTTCTTGCCGCCGTTGCTGCCCTTGCCGT	+418 (as)	RT-qPCR
pgx6-1	GAAGTCATCGCAAGGTCTATAC	+136 (s)	RT-qPCR
pgx6-2	AGAACAGAATAGGTCGGAGGTA	+635 (as)	RT-qPCR
**HYGROMYCIN CASSETTE**			
hyg-G	CGTTGCAAGACCTGCCTGAA	+289 (s)	Split marker
hyg-Y	GGATGCCTCCGCTCGAAGTA	+735 (as)	Split marker
TtrpC-8B	AAACAAGTGTACCTGTGCATTC	+1727 (as)	Split marker
FOXG_08883-10	agagataactccccgagcatcGGAGAGACGGACGGACGCA	−675 (s)	Split marker
**PHLEOMYCIN CASSETTE**			
TtrpC-8B	AAACAAGTGTACCTGTGCATTC	+1133 (as)	Site-directed mutagenesis
PgpdA-15B	CGAGACCTAATACAGCCCCT	−1193 (s)	Site-directed mutagenesis
**ACTIN**			
act-q7	ATGTCACCACCTTCAACTCCA	+1278 (s)	RT-qPCR
act-q8	CTCTCGTCGTACTCCTGCTT	+1578 (as)	RT-qPCR

*Lowercase is the overlapping region with 3′ end of the Hyg^R^ cassette to generate DNA fragment fusions by PCR amplification. Positions are referred to the start codon: (+) downstream, (−) upstream of the ATG. s, Sense; as, antisense*.

### Biochemical enzyme characterization assays

PG activity was screened on SM plates as described previously (Scott-Craig et al., 1990) with minor modifications. Plates containing 1% (w/v) PGA, 1.5 M (NH_4_)_2_SO_4_, and 14% (w/v) MES (Sigma-Aldrich, Germany) were adjusted to pH 5.2 with NaOH. For total PG or PL activity, the wild type and mutant strains were induced on 0.5% pectin for different time periods. Cultures were filtered through nylon monodur (pore size 10 nm) to separate and discard the mycelia. The filtrate was centrifuged and the supernatant transferred to dialysis tubing (12 kDa cut-off), dialyzed overnight against distilled water and concentrated 50-fold by placing the tubing on solid polyethylene glycol (35 k Mr; Fluka Chemika-Biochemika, Switzerland). Aliquots from induced supernatants were used as enzyme source for PG or PL activity assays. Protein quantities were determined by using the BioRad protein assay (BioRad, Hercules, CA) with bovine serum albumin as standard. Total PG activity was determined by measuring the release of reducing groups from polygalacturonic acid (PGA) using the method of Nelson-Somogyi as described previously (Di Pietro and Roncero, 1996). Appropriate controls without either enzyme or substrate were run simultaneously. The quantity of reducing sugar released was calculated from standards of D-galacturonic acid. Enzyme activity is expressed in μg of D-galacturonic acid released per min and μg of protein used. Each assay was carried out three times in 96-well microtiter plates. Total PL activity was determined by measuring the release of reducing sugar from PGA using the method described previously (Muslim et al., 2015) with some modifications. Significance of activity data was calculated by student's *t*-distribution statistic test (> 0.05).

### Expression analyses of polygalacturonase, xylanase, and pectate lyase genes

Expression patterns of genes encoding the two major PGs (FOXG_14695, *pg1*, and FOXG_15415, *pgx6*) (Bravo-Ruiz et al., 2016), a pectate lyase gene (FOXG_12264, *pl1*) (Huertas-González et al., 1999) and two xylanases (FOXG_13415, *xyl3*, and FOXG_15742, *xyl4*) (Gómez-Gómez et al., 2002), were performed under pectin or TVT induction (see above), or under glucose repression conditions. RNA from germlings grown 4 h on 0.5% pectin, on 0.5% pectin plus 0.5% glucose; or on 2.5% TVT for 4 and 24 h, was analyzed by quantitative RT-qPCR. Quality of extracted nucleic acids was verified by running aliquots in ethidium bromide stained agarose gels (0.7% w/v in TAE buffer) and further visualized under UV light. Additionally, they were quantified in a NanoDrop ND-1000 Spectrophotometer. The isolated RNA was treated with Desoxyribonuclease I (DNase I, Fermentas, USA) and then used to synthesize cDNA using the Transcriptor Universal cDNA Master (Roche Life Sciences), according to the manufacturer instructions. Quality of extracted nucleic acids was verified by running aliquots in ethidium bromide stained agarose gels (0.7% w/v in TAE buffer) and further visualized under UV light. Additionally, they were quantified in a NanoDrop ND-1000 Spectrophotometer. Total cDNA obtained was subject to RT-qPCR using the appropriate gene-specific primer pairs (see Table 2 for primers). Three simultaneous technical replicate amplifications were carried out for each cDNA sample. Amplification reactions were performed in 96-well microtiter plates (BioRad, Hercules, USA) and each reaction was made up using aliquots from the same master mix. PCRs were performed in an iCycler iQ5 real-time PCR System (BioRad, Hercules, USA) using the following cycling protocol: an initial step of denaturation (5 min, 94°C) followed by 40 cycles of 30 s at 94°C, 30 s at 62°C, 45 s at 72°C, and 20 s at 80°C for measurement of the fluorescence emission. After this, a melting curve program was run for which measurements were made at 0.5°C temperature increases every 5 s within a range of 55–95°C. Relative levels of the RT-qPCR products were determined using the ΔΔCt method (Livak and Schmittgen, 2001). Expression values are presented as relative to those of wild type strain and normalized to the Ct value of the actin gene (Table 2). Samples without cDNA were used as negative control reactions. The assay was repeated with three independent biological replicates.

### Virulence assays

Root inoculation assays were performed as described previously (Di Pietro and Roncero, 1998). Briefly, 14-day-old tomato seedlings (cultivar Monika, seeds kindly provided by Syngenta Seeds, Almeria, Spain) were inoculated with *F. oxysporum* f. sp. *lycopersici* strains by immersing the roots in a suspension of 5 × 10^6^ microconidia mL^−1^ for 30 min, planted in vermiculite and maintained in a growth chamber. Ten plants were used for each treatment (Di Pietro and Roncero, 1998). Severity of disease symptoms and plant survival was recorded daily for 35 days as previously described (López-Berges et al., 2010). Virulence experiments were performed at least three times with similar results. Plant survival was calculated by the Kaplan-Meier method and compared among groups using the log-rank test. Data were analyzed with the software GraphPad Prism 4.

## Results and discussion

### Comparative genome analysis of *P. occitanis*

The genomes of the wild type strain and the hyperpectinolytic mutant of *P. occitanis* were sequenced, assembled and annotated (see Materials and Methods). The resulting assemblies comprised roughly 1,600 contigs, 80 of which were larger than 100 kb (Supplementary Table 1), the total estimated genome size for both strains was of 36.3 Mb. Compared to its closely related, fully sequenced, species, *P. occitanis* is roughly the same size as its two closest relatives *Talaromyces cellulolyticus* (36.2 Mb) and *T. verruculosus* (36.7 Mb) and much larger than the human pathogen *Penicillium marneffei* (28.7 Mb). The gene prediction showed that *P. occitanis* encoded between 11,233 and 11,269 proteins, which is a bit higher than its closest sequenced relative *T. cellulolyticus* (10,910) but within normal parameters for *Talaromyces* species (from 10,001 in *P. marneffei* to 12,996 in *T. stipitatus*). The phylome, the complete collection of phylogenetic trees of each gene encoded in the *P. occitanis* genome was reconstructed (see Materials and Methods). Each individual gene tree was automatically analyzed with phylogeny-based algorithms that predict orthology and paralogy relationships (Gabaldón, 2008), and detect and date duplication events (Huerta-Cepas and Gabaldón, 2011). The reconstructed trees, alignments and orthology and paralogy predictions are accessible to browse or download at the PhylomeDB database (Huerta-Cepas et al., 2014). 505 one-to-one orthologs present across the 29 considered species (Supplementary Table 2) were used to reconstruct a species tree using an alignment concatenation approach (see Materials and Methods).

As seen in Figure 1, *P. occitanis* groups with the *Talaromyces* species and not with the other *Penicillium* species. Therefore, *P. occitanis* should likely be renamed to reflect its correct phylogenetic position within the Talaromyces clade. To avoid confusion with previously published literature, we decided to maintain the name *P. occitanis* in this work. Within the *Talaromyces* group, *P. occitanis* is more closely related to *Talaromyces cellulolyticus*. Of the predicted proteins, only 98 were found to be orphan genes. Analysis of the gene trees reconstructed in the phylome also revealed few species specific expansions in *P. occitanis*, which is consistent with the overall low duplication rates—i.e., the number of duplications per branch per gene—found in *Talaromyces* species.

We provide the first genome sequence for *P. occitanis* and a first annotation and comparative analysis for it. The genome sequence, annotation, and the extensive genome-wide phylogenetic analysis constitute important resources that will certainly pave the way for a better understanding of the evolution of this species and the processes in which it is involved. Our results suggest that *P. occitanis* contains a large number genes involved in pectin and xyloglucan degradation. Sequencing of a *P. occitanis* hyperpectinolytic strain obtained through mutagenesis, reveals a small set of point mutations likely to contain the one responsible for the phenotypic change. Our best candidate consisted in a non-synonymous mutation affecting a conserved residue of the Crlb-like transcription factor.

### *P. occitanis* as a producer of carbohydrate catabolic enzymes

Hydrolyzing enzymes have been categorized within the Carbohydrate-Active enZymes database and are known as CAZy proteins. *P. occitanis* was obtained from soil and mutants were created in an attempt to enhance its natural capacity to produce enzymes able to degrade polysaccharides. The availability of the whole genome of *P. occitanis* now allows for the search of the genes responsible for the degradation of these compounds. We used dbCan (Yin et al., 2012) in order to assign CAZy domains to the proteins of the 29 considered species (Supplementary Table 2). 692 CAZy proteins are encoded in the *P. occitanis* genome. The two closest related species to *P. occitanis* have a similar number of CAZy proteins (*T. cellulolyticus*—694 and *T. verruculosus*—685), which is higher than that of the other *Talaromyces* species (*Talaromyces islandicus*–599). In fact, the first three species contain the highest number of CAZy proteins out of the entire set of *Eurotiomycetes* species considered in this study. Only the genomes of the three *Fusarium* species encode a higher number of CAZy proteins [*F. gramineraum* (873), *F. oxysporum* (837) and *F. verticillioides* (729)]. In relative terms, however, the genomes of the first three *Talaromyces* species, including *P. occitanis*, have the highest percentage of genes encoding CAZy proteins (ranging from 6.04 to 6.36%). *Fusarium* species, with larger genomes, have a smaller percentage of their genome encoding for CAZy proteins (5.07% on average).

CAZy proteins are subdivided into different families and we compared the number of proteins each species had grouped in each family (Supplementary Table 3). There do not seem to be any major differences between the three *Talaromyces* species, which could indicate that the increase in CAZy proteins pre-dates the divergence of these three species. When compared to the other species included in the analysis, with a particular focus on the *Fusarium* species that also have a large amount of CAZy proteins, we noticed that the increase in CAZy proteins was spread out among the different CAZy categories and not due to the expansion of a specific protein family.

Some CAZy families have been associated to the degradation of specific compounds (Espagne et al., 2008; Amselem et al., 2011; van den Brink and de Vries, 2011; Benoit et al., 2012). These families are not necessarily specific, some of them have been associated to more than one compound. According to this classification, *P. occitanis* has 158 proteins associated to pectin degradation (Supplementary Table 4). This value is only found increased in some *Fusarium* species, with *Nectria haematococca* having the highest number of pectin degrading proteins (217). Curiously, *P. occitanis* seems to have an increased capability to degrade xyloglucan as compared to the other species, at least in terms of the number of encoded proteins associated to the degradation of this compound. As before, this trend is shared with the two most closely related *Talaromyces* species, *T. verruculosus* and *T. cellulolyticus*. With an average of 68 proteins that belong to this category, it more than doubles the average number of proteins in the other species considered (average of 32 proteins).

### A mutation in CLrB as the possible cause for hyperpectinolytic activity in *P. occitanis* CT1

**Figure 2:**
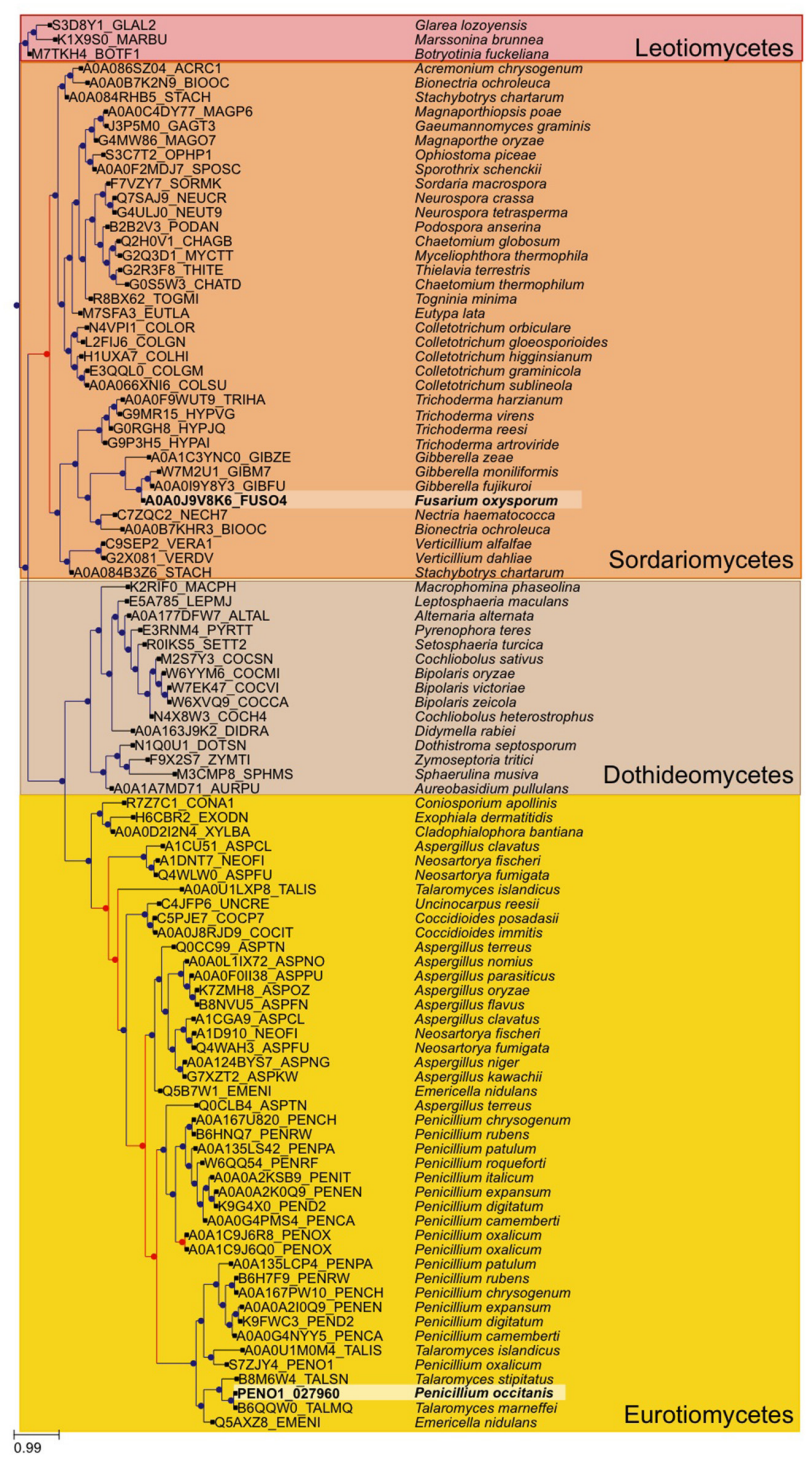
Gene tree of the Crlb-like transcription factor found to have a mutation in the *P. occitanis* mutant. The tree was reconstructed by performing a blastp search against UniProt. The sequences were downloaded and the tree was reconstructed using the same pipeline used in the phylome. Sequences in bold and surrounded by a lighter square belong to the *P. occtanis* transcription factor and its ortholog in *F. oxysporum*. Duplication nodes are marked in red and speciation nodes are marked in blue according to the species overlap algorithm.

The two strains of *P. occitanis* were compared in order to search for the mutation that caused *P. occitanis* CT1 to be able to secrete large amounts of pectinases (Supplementary Tables 5, 6). Seven reliable SNPs were detected comparing the two strains that were associated to proteins (see Materials and Methods, and Table 1). Four of these were located in putative promotor regions (i.e., 1,000 bp upstream from a protein-coding gene). One other SNP caused a synonymous mutation within a protein and two others were found to cause non-synonymous mutations (Table 1). Given the observed deregulation of pectinolytic enzymes observed in the mutant strain, we focused our attention in a non-synonymous SNPs affecting a transcription factor (ortholog to *Aspergillus nidulans* ClrB), which results in a Serine to Cysteine mutation at position 198 of the amino acid sequence. To broaden the taxonomic scope considered in the phylome, we reconstructed an additional phylogenetic tree after running a blast search against UniProt and using the same tree reconstruction pipeline used during the phylome reconstruction (Figure 2). The tree shows that this transcription factor is very conserved among *Pezizomycotina* species. The alignment showed that the mutation had occurred in a very conserved position in the alignment where nearly all the other *Pezizomycotina* species have a serine in position 198. The only exceptions are three sequences within *Sordariomycetes* that have a S → G mutation and a sequence in *A. terreus* that has a S → A mutation. None of the sequences found have the same mutation found in *P. occitanis* CT1.

Ayadi et al. (2011) had previously hypothesized that the hyperpectinolytic activity of CT1 could be the result of a mutation in a transcription factor, due to the fact that many different proteins appeared to be activated. In *Neurospora crassa* and *A. nidulans*, two zinc binuclear cluster transcription factors Clr-1, Clr-2, and ClrA, ClrB, respectively, were reported to be required for normal growth and enzymatic activity on cellulose or cellobiose, but not on xylan (Coradetti et al., 2012). Enzyme activity was abolished in *A. nidulans* Δ*clrB* mutant, while the Δ*clrA* mutant showed ~50% of wt activity. Consistent with this, induction of major cellulase genes in the Δ*clrB* mutant was 1,000-fold reduced compared to the wt, while in the Δ*clrA* mutant, the average induction was 2-to 4-fold less. However, the role of these transcription factors in growth on pectin has not been tested so far (Coradetti et al., 2013).

### Primary structure of the FOXG_08883 gene

**Figure 3:**
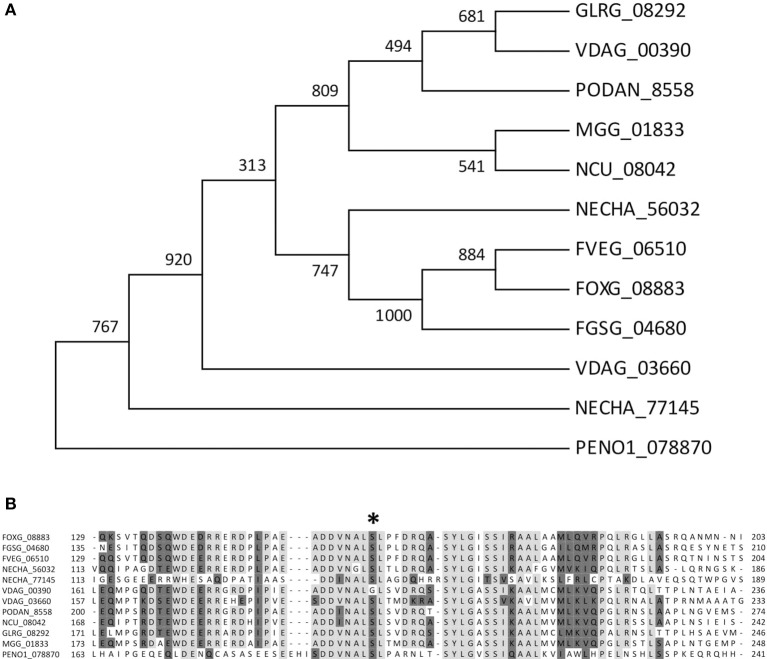
Multiple sequence alignment of *F. oxysporum* predicted Zn_2_Cys_6_ transcription factor FOXG_08883 with orthologs from related fungal species *Fusarium graminearum, Fusarium verticillioides, Penicillium occitanis, Nectria haematococca, Verticillium dhaliae, Podospora anserina, Neurospora crassa, Colletotrichum graminicola*. **(A)** Maximum Likelihood phylogenetic tree depicting the evolutionary relationship of FOXG_08883 and its homologs in other fungal species. **(B)** Alignment of deduced amino acids located in a very conserved region involving the position 198^Ser^ to 198^Cys^ (asterisk) found in PENO1_078870 gene.

To functionally test the role of the predicted candidate gene in regulation of pectinolytic enzymes, we chose *F. oxysporum* for two main reasons. First, the availability of an efficient transformation system, together with the biochemical and molecular biology tools which are not available in *P. occitanis* or *Penicillium expansum*; second, the interest in the identification of CWDE regulatory mechanisms and their relevance during plant infection. A blastp search of the *F. oxysporum* genome database as well as a phylome analysis identified the gene FOXG_08883 as the predicted ortholog of the *P. occitanis* PENO1_078870 gene. The amino acid sequence of the FOXG_08883 product shows significant similarity to transcription factors from related fungal species (Figures 3A,B) and encodes for a putative protein of 709 amino acids containing a characteristic GAL4-like Zn_2_Cys_6_ binuclear cluster DNA-binding domain and the fungal transcription factor regulatory middle homology region, suggesting a possible function as a transcription factor. The 5‘ upstream region of FOXG_08883 contained a number of motifs, some of which correspond to sequences present as repeats in the *F. oxysporum* genome, including two consecutive regions of 623 and 362 bp, respectively, which are present 226 and 214 times, respectively (Supplementary Figure 1A).

### Construction of mutants in the FOXG_08883 gene

Targeted gene deletion followed by site-directed mutagenesis was performed to replace the wild type FOXG_08883 allele by the ^Cys^160^Ser^ mutant allele in the *F. oxysporum* knock-out background. Targeted deletion of FOXG_08883 was initially achieved by replacing the complete ORF with the hygromycin resistance cassette (Supplementary Figure 1B). Transformants were analyzed by Southern analyses and PCR (Supplementary Figures 1C,D). Two transformants (named FOXG_08883Δ10 and FOXG_08883Δ11) lacked the wild type hybridization bands, suggesting gene deletion, but only FOXG_08883Δ11 showed amplification bands corresponding to promoter and terminator regions with primers located outside the construction, and failed to amplify the ORF. Generation of site-directed mutants was performed by co-transformation of the FOXG_08883Δ11 strain with the phleomycin resistance cassette and a point-mutated FOXG_08883 allele in which cytosine^599^ had been replaced with a guanine and adenine^600^ with a thymine, thus originating a Ser by Cys change at position 160 of the encoded protein. Transformants carrying the mutated allele were confirmed by PCR with specific primers of the FOXG_08883 ORF (Supplementary Figure 2A) and sequencing of the resulting fragments. Southern blot analyses confirmed that nine transformants harbored the correctly mutated allele (Supplementary Figure 2B). Two mutants were selected for further studies: M50 with a unique integration and M22 with two copies of the mutated version.

### Biochemical and phenotypic characterization of FOXG_08883 mutants

**Figure 4:**
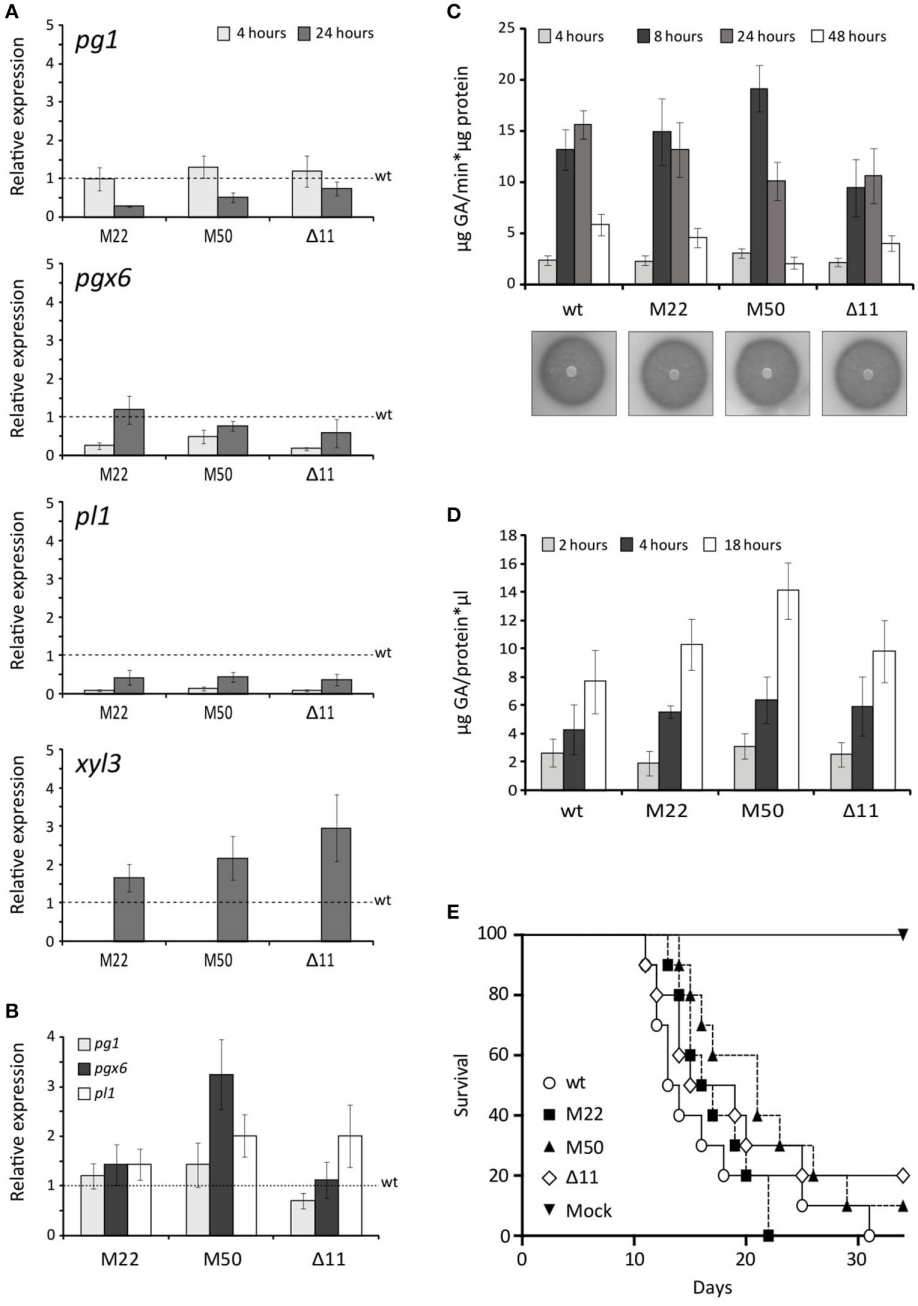
Phenotypic characterisation of FOXG_08883 mutants. **(A)** Transcript levels of *pg1, pgx6, pl1* and *xyl3* genes were measured by RT-qPCR, in mutants and wild type strains (wt), grown for 4 and 24 h on 2.5% tomato vascular tissue (TVT). Bars represent standard errors calculated from three biological replicates each including three technical replicates **(B)** Transcript levels of *pg1, pgx6* and *pl1* genes were measured by qRT-PCR in *F. oxysporum* mutants compared wild type strain (wt), under 0.5% pectin-induced conditions for 4 h. Bars represent standard deviations calculated from three biological replicates each including three technical replicates **(C)** PG activity of the indicated strains was determined by halo formation on PGA-containing plates, and as total specific activity, from culture filtrates after 4, 8, 24, and 48 h growth on SM supplemented with 0.5% pectin, determined by Nelson-Somogyi method using 1% PGA as substrate. Enzyme activity is expressed as μg D-galacturonic acid released per min and μg of protein. Bars represent standard errors calculated from three biological replicates each including two technical replicates **(D)** Total PL activity in culture filtrates after 24 h growth on SM supplemented with 0.5% pectin, determined by measuring absorbance at 230 nm after incubation at 37°C for different times (2, 4, 18 h) in 50 mM Tris-HCl pH 8 buffer containing 1 mM of calcium chloride and using 2% PGA as substrate. Enzyme activity is expressed as μg of GA released per μl of sample and μg of protein. Bars represent standard errors calculated from three biological replicates each with two technical replicates **(E)** Incidence of *Fusarium* wilt of tomato plants (cultivar Monika) caused by the wild type strain (wt) and the different mutants. Groups of 10 plants were inoculated by immersing the roots into a suspension of 5 × 10^6^ freshly obtained microconidia mL^−1^ from each strain and planted in minipots. Percentage survival was recorded daily. Data shown are from one representative experiment. Experiments were performed three times with similar results.

To investigate the implication of FOX_08883 in regulation of polygalacturonase (*pg*), pectate lyase (*pl*) or xylanase (*xyl*) gene expression, RT-qPCR analysis was performed using RNA extracted from *F. oxysporum* germlings incubated in SM supplemented with 2.5% (w/v) tomato vascular tissue (TVT) (Figure 4A), 0.5% (w/v) pectin (Figure 4B) or 0.5% (w/v) pectin plus 0.5% (w/v) glucose (data not shown). Under TVT induction, the different mutants showed a 30–70% reduced expression, compared to the wild type strain, of *pg1* after 24 h and 50–80% of *pgx6* after 4 h. The decrease in expression was more severe in the case of *pl1*, which was 90% reduced after 4 h and 50% after 24 h. In contrast, the mutants showed increased expression for *xyl3*, whereas *xyl4* transcripts were unaffected in all conditions (data not shown). In the presence of pectin, neither the deletion nor the point-mutation mutants of FOXG_08883 were significantly affected in gene expression. As previously shown, glucose represses expression of all PG genes (Di Pietro and Roncero, 1998; Garcia-Maceira et al., 2000, 2001; Bravo-Ruiz et al., 2016). No difference in carbon catabolite repression was detected in both types of mutants (data not shown). In addition, PG and PL activities on PGA plates and in culture supernatants of pectin cultures were analyzed to determine the effect of FOXG_08883 deletion on secretion of pectinolytic enzymes. In no case significant differences between mutant and wild type strains were observed (Figures 4C,D).

A *N. crassa* Δ*clr-1* mutant was found to grow poorly on cellobiose (Coradetti et al., 2012, 2013). By contrast, the *F. oxysporum* ΔFOXG_08883 mutant showed no difference in growth on solid or liquid minimal media containing either carboxymethylcellulose, cellobiose or cellulose as sole carbon source (Supplementary Figure 3 and Supplementary Table 7). This points to differences in the regulatory mechanisms of cellulase genes between different *Pezizomycotina* species. Although our attempts to reproduce the phenotypic change in *F. oxysporum* by mutating the equivalent residue in the orthologous protein have failed, this mutation remains our best candidate. Indeed the two species are rather distantly related and, although *P. occitanis* only contains one copy of the gene, the phylogenetic analysis (Figure 2) uncovers a duplication preceding the divergence of *Talaromyces* and *Penicillium* species, followed by a loss of one of the duplicates in the former group. Thus, it is likely that the two orthologues may not fulfill fully equivalent functions.

### Role of FOXG_08883 in virulence on tomato plants

The role of the putative transcription factor encoded by FOXG_08883 in virulence was determined by inoculating 2-week-old tomato plant roots in microconidial suspensions of the wild type strain, the FOXG_08883Δ11 mutant and the two Δ11::FOXG_08883^160Ser>Cys^ mutants (M22 and M50). Plants were scored for vascular wilt symptoms at different time intervals after inoculation. Severity of wilt symptoms in plants inoculated increased steadily throughout the experiment, leading to characteristic wilt symptoms 8 days after inoculation, and most plants were dead 30 days after inoculation, without significant differences between the mutants and the wild type strain (Figure 4E).

The availability, annotation and extensive phylogenomic analysis of the *P. occitanis* genome sequence represents an important resource for understanding the evolution and biology of this species, and sets the basis for the discovery of new genes for the degradation of complex polysaccharides, of high biotechnological interest.

## Availability of data and materials

All the materials described in the manuscript, including all relevant raw data, are freely available to any scientist wishing to use them for non-commercial purposes, without breaching participant confidentiality. The final annotation comprised 11,269 protein-coding genes. This Whole Genome Shotgun project has been deposited at DDBJ/ENA/GenBank under the accession NPFJ00000000 (*Penicillium occitanis* CT1), and NPFK00000000 (*Penicillium occitanis* CL100). The version described in this paper is version NPFJ01000000.

## Author contributions

GB and AH performed experimental work, MM conducted bioinformatic analyses. MR, AG, and AD conceived the study. TG and MR coordinated the work, analyzed the data and drafted the manuscript. All authors read and approved the final manuscript.

### Conflict of interest statement

The authors declare that the research was conducted in the absence of any commercial or financial relationships that could be construed as a potential conflict of interest.

## Supplementary material

Supplementary Figure 1 Strategy for targeted gene deletion **(A)** Physical structure of FOXG_08883 gene promoter and coding regions. Different domains found within the 5′UTR are boxed indicating their length and number of repeats found within *F. oxysporum* genome as well as relative location to the ATG codon. The presumed GAL4-like Zn_2_Cys_6_ binuclear cluster DNA-binding domains and the fungal transcription factor (TF) regulatory middle homology region are indicated within the coding region **(B)** Strategy for targeted replacement of the ORF using the split marker technique and the hygromycin cassette (Hyg^R^) as selective marker. Primers used for amplification of both overlapping fragments or for confirmation of targeted gene-replacement are indicated with black arrows (sense) or light arrows (antisense). Primer names are simplified by their identification numbers. DNA fragments used as probes for Southern analyses with restriction sites *Hind* III and *Eco* RI are shown. Scale bar indicates 1 kb length **(C)** Southern analyses of wild type (wt) and targeted deletion strains (Δ), or ectopic transformants (Ect). gDNAs were treated with *Hind* III and *Eco* RI and hybridized with the FOXG_08883 ORF probe **(D)** PCR confirmation of targeted mutants using gDNAs as template and the indicated primers.

Supplementary Figure 2 Strategy for generation of site-directed mutants **(A)** Co-transformation of the FOXG_08883Δ11 mutant with a FOXG_08883^160Ser>Cys^ allele and the phleomycin cassette (Phl^R^). DNA fragments used as probes for Southern analyses, primers used, and restriction sites for *Eco* RI are shown. Scale bar indicates 1 kb **(B)** Southern analysis of wild type (wt), deletion mutant FOXG_08883Δ11, and different Phl^R^ transformants. gDNAs were treated with *Eco* RI and hybridized with the FOXG_08883 ORF as probe.

Supplementary Figure 3 Fungal colonies from microconidia serial dilutions of the wild type (wt) and the indicated mutant strains grown for 3 days at 28°C on synthetic media (SM) containing the indicated carbon source: Sucrose, Cellobiose, or Carboxymethyl (CM)-cellullose.

Supplementary Table 1 Genome characteristics for the assembly of the two strains.

Supplementary Table 2 List of species included in the three phylomes.

Supplementary Table 3 Number of homologs per species for each CAZY family.

Supplementary Table 4 Number of proteins per species that degrades each compound.

Supplementary Table 5 Mutations in PENOC when compared to the base genome PENO1, only mutations that affect a protein.

Supplementary Table 6 Mutations in PENOC when compared to the base genome PENO1, only mutations that affect a protein.

Supplementary Table 7 Mycelium dry weight (mg) after 120 h growth in SM containing 0.5% of the indicated carbon source, with 170 rpm at 28°C.
