# Supplementary figures and images for: Regulatory Mechanisms of a Highly Pectinolytic Mutant of Penicillium occitanis and Functional Analysis of a Candidate Gene in the Plant Pathogen Fusarium oxysporum
Source: Front Microbiol. 2017 Sep 8;8:1627. doi: 10.3389/fmicb.2017.01627 (PMC5599776; doi:10.3389/fmicb.2017.01627)

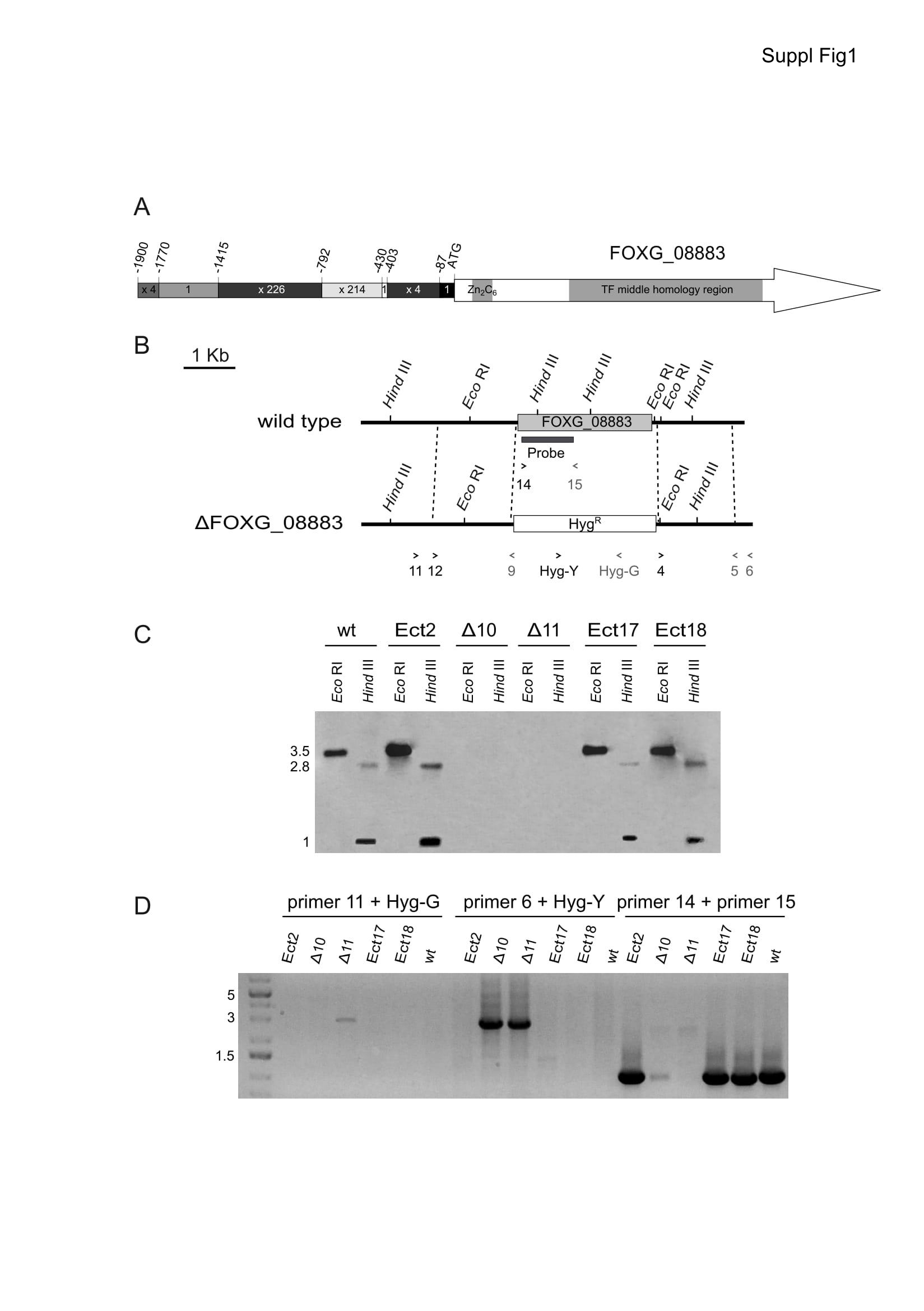

Supplement: Supplementary Figure 1 — Strategy for targeted gene deletion (A) Physical structure of FOXG_08883 gene promoter and coding regions. Different domains found within the 5′UTR are boxed indicating their length and number of repeats found within F. oxysporum genome as well as relative location to the ATG codon. The presumed GAL4-like Zn2Cys6 binuclear cluster DNA-binding domains and the fungal transcription factor (TF) regulatory middle homology region are indicated within the coding region (B) Strategy for targeted replacement of the ORF using the split marker technique and the hygromycin cassette (HygR) as selective marker. Primers used for amplification of both overlapping fragments or for confirmation of targeted gene-replacement are indicated with black arrows (sense) or light arrows (antisense). Primer names are simplified by their identification numbers. DNA fragments used as probes for Southern analyses with restriction sites Hind III and Eco RI are shown. Scale bar indicates 1 kb length (C) Southern analyses of wild type (wt) and targeted deletion strains (Δ), or ectopic transformants (Ect). gDNAs were treated with Hind III and Eco RI and hybridized with the FOXG_08883 ORF probe (D) PCR confirmation of targeted mutants using gDNAs as template and the indicated primers. [file Image1.PNG]

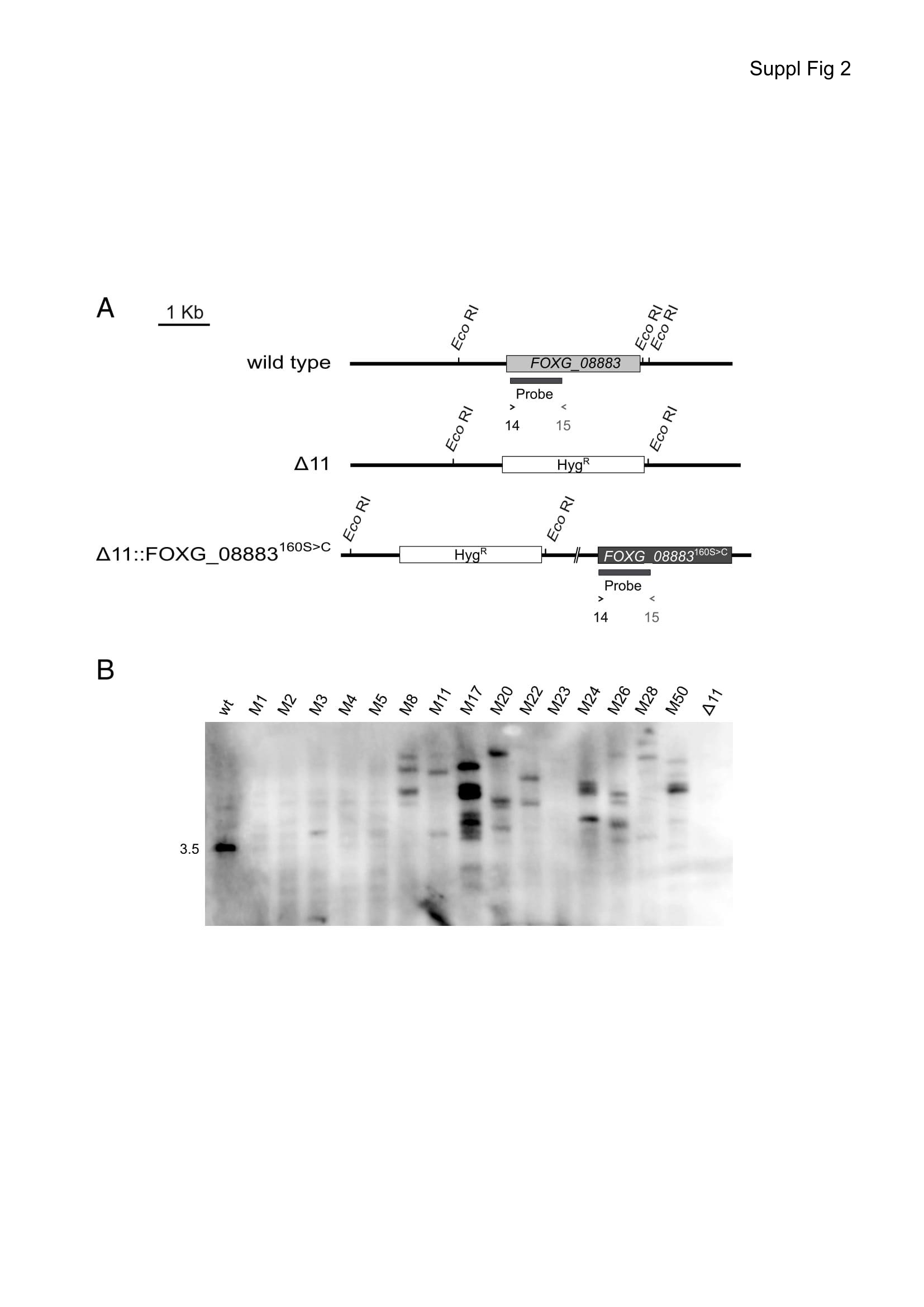

Supplement: Supplementary Figure 2 — Strategy for generation of site-directed mutants (A) Co-transformation of the FOXG_08883Δ11 mutant with a FOXG_08883160Ser>Cys allele and the phleomycin cassette (PhlR). DNA fragments used as probes for Southern analyses, primers used, and restriction sites for Eco RI are shown. Scale bar indicates 1 kb (B) Southern analysis of wild type (wt), deletion mutant FOXG_08883Δ11, and different PhlR transformants. gDNAs were treated with Eco RI and hybridized with the FOXG_08883 ORF as probe. [file Image2.PNG]

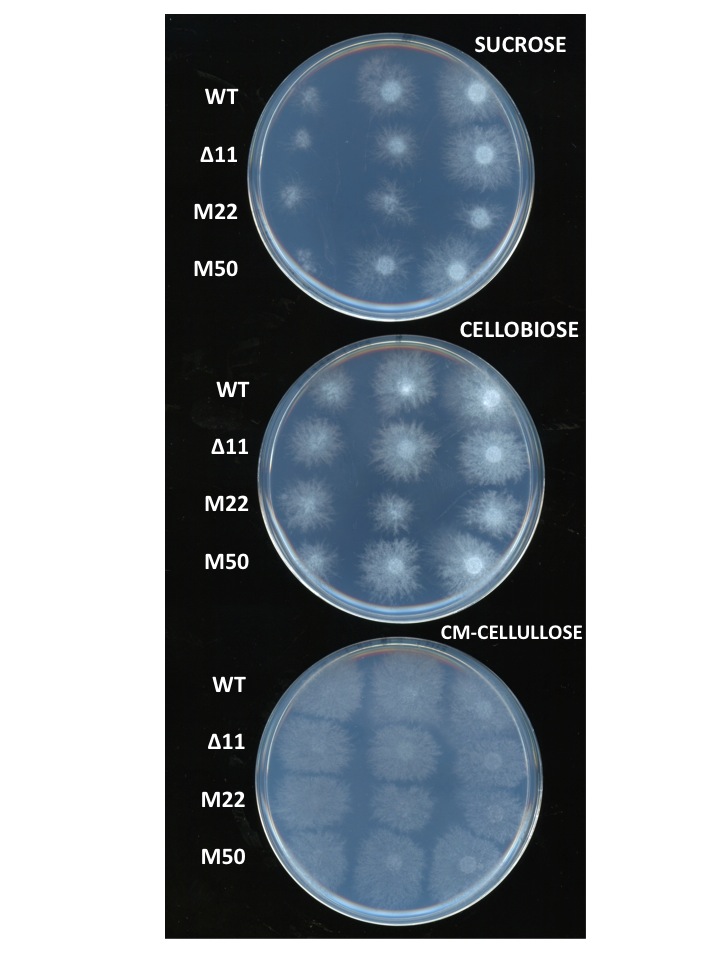

Supplement: Supplementary Figure 3 — Fungal colonies from microconidia serial dilutions of the wild type (wt) and the indicated mutant strains grown for 3 days at 28°C on synthetic media (SM) containing the indicated carbon source: Sucrose, Cellobiose, or Carboxymethyl (CM)-cellullose. [file Image3.JPEG]
