# Supplementary material for: Regulatory Mechanisms of a Highly Pectinolytic Mutant of Penicillium occitanis and Functional Analysis of a Candidate Gene in the Plant Pathogen Fusarium oxysporum
Source: Front Microbiol. 2017 Sep 8;8:1627. doi: 10.3389/fmicb.2017.01627 (PMC5599776; doi:10.3389/fmicb.2017.01627)
Supplement: Supplementary Table 1 — Genome characteristics for the assembly of the two strains. [file Table1.DOCX]

**Supplementary Table** **1** Genome characteristics for the assembly of the two strains.

|  | ***P. occitanis CL100*** | ***P. occitanis CT1*** |
| --- | --- | --- |
| Genome code | PENO1 | PENOC |
| Genome size | 36.3 Mb | 36.4 Mb |
| Number of contigs | 1592 | 1651 |
| Number of contigs > 100 kb | 77 | 82 |
| N50 | 67 Kb | 67 Kb |
| GC content | 0.47 | 0.47 |
| Number of proteins | 11233 | 11269 |
| Percentage of genes with introns | 77 % | 77 % |
| Average number of exons | 3 | 3 |
| Average protein length | 512 aa | 512 aa |
