# Supplementary material for: Regulatory Mechanisms of a Highly Pectinolytic Mutant of Penicillium occitanis and Functional Analysis of a Candidate Gene in the Plant Pathogen Fusarium oxysporum
Source: Front Microbiol. 2017 Sep 8;8:1627. doi: 10.3389/fmicb.2017.01627 (PMC5599776; doi:10.3389/fmicb.2017.01627)
Supplement: Supplementary Table 2 — List of species included in the three phylomes. [file Table2.DOCX]

**Supplementary Table 2** List of species included in the three phylomes.

| **TaxID** | **Species name** | **Source** |
| --- | --- | --- |
| 5037 | *Ajellomyces capsulata* | Broad Institute |
| 330879 | *Aspergillus fumigatus* | UniProt |
| 5061 | *Aspergillus niger* | UniProt |
| 40559 | *Botrytis fuckeliana* | Broad Institute |
| 1356009 | *Byssochlamys spectabilis No. 5* | UniProt |
| 5501 | *Coccidioides immitis* | Broad Institute |
| 162425 | *Emericella nidulans* | UniProt |
| 1263415 | *Endocarpon pusillum Z07020* | UniProt |
| 5507 | *Fusarium oxysporum* | Broad Institute |
| 1028729 | *Fusarium pseudograminearum* | UniProt |
| 117187 | *Gibberella moniliformis* | Broad Institute |
| 5518 | *Gibberella zeae* | Broad Institute |
| 1047171 | *Mycosphaerella graminicola* | JGI |
| 140110 | *Nectria haematococca* | JGI |
| 5076 | *Penicillium chrysogenum* | Hyphal tip |
| 27334 | *Penicillium expansum* | CRG genome annotation |
| 441960 | *Penicillium marneffei ATCC 18224* | Hyphal tip |
| 290292 | *Penicillium occitanis* | CRG genome annotation |
| 933388 | *Penicillium oxalicum 114-2* | NCBI |
| 321614 | *Phaeosphaeria nodorum SN15* | Broad Institute |
| 284812 | *Schizosaccharomyces pombe (strain 972/ATCC 24843)* | Quest for orthologs |
| 5180 | *Sclerotinia sclerotiorum* | Broad Institute |
| 1472165 | *Talaromyces cellulolyticus* | NCBI |
| 28573 | *Talaromyces islandicus* | NCBI |
| 441959 | *Talaromyces stipitatus ATCC 10500* | NCBI |
| 198730 | *Talaromyces verruculosus* | NCBI |
| 63577 | *Trichoderma atroviride* | JGI |
| 51453 | *Trichoderma reesei* | JGI |
| 29875 | *Trichoderma virens* | JGI |
