# Supplementary material for: Regulatory Mechanisms of a Highly Pectinolytic Mutant of Penicillium occitanis and Functional Analysis of a Candidate Gene in the Plant Pathogen Fusarium oxysporum
Source: Front Microbiol. 2017 Sep 8;8:1627. doi: 10.3389/fmicb.2017.01627 (PMC5599776; doi:10.3389/fmicb.2017.01627)
Supplement: Supplementary Table 5 — Mutations in PENOC when compared to the base genome PENO1, only mutations that affect a protein. [file Table5.DOCX]

**Supplementary Table 5** Mutations in PENOC when compared to the base genome PENO1, only

**mutations that affect a protein.**

| **Contig** | **Position** | **Change** | **Kind of mutation** | **Protein affected** |
| --- | --- | --- | --- | --- |
| contig_17 | 113805 | G → A | Non-coding region | 99 bp upstream of PENO1_011980 |
| contig_32 | 110697 | C → A | Non-coding region | 134 bp upstream of PENO1_019200 |
| contig_55 | 40891 | C → G | Non-synonymous mutation | PENO1_027960 (198S → 198C) |
| contig_58 | 80023 | T → C | Synonymous mutation | PENO1_029210 |
| contig_63 | 84736 | A → G | Non-coding region | 672 bp upstream of PENO1_031020 |
| contig_172 | 6129 | A → G | Non-synonymous mutation | PENO1_060250 (620S → 620G) |
| contig_218 | 28876 | C → A | Non-synonymous mutation | PENO1_069310 (206G → 206*) |
| contig_388 | 1499 | A → T | Non-synonymous mutation | PENO1_091170 (298E → 298V) |
| contig_723 | 2385 | A → G | Synonymous mutation | PENO1_107960 |
| contig_725 | 764 | C → G | Non-coding region | 696 bp upstream of PENO1_108040 |
| contig_749 | 6272 | C → T | Non-coding region | 913bp upstream of PENO1_108560 |
