# Supplementary material for: Regulatory Mechanisms of a Highly Pectinolytic Mutant of Penicillium occitanis and Functional Analysis of a Candidate Gene in the Plant Pathogen Fusarium oxysporum
Source: Front Microbiol. 2017 Sep 8;8:1627. doi: 10.3389/fmicb.2017.01627 (PMC5599776; doi:10.3389/fmicb.2017.01627)
Supplement: Supplementary Table 6 — Mutations in PENOC when compared to the base genome PENO1, only mutations that affect a protein. [file Table6.DOCX]

**Supplementary Table 6 Mutations in PENOC when compared to the base genome PENO1, only mutations that affect a protein.**

| **Contig** | **Position** | **Change** | **Kind of mutation** | **Protein affected** |
| --- | --- | --- | --- | --- |
| contig_120 | 59551 | A → C | Non-coding region | 134 bp upstream of PENOC_048850 |
| contig_17 | 113798 | A → G | Non-coding region | 99 bp upstream of PENOC_010810 |
| contig_229 | 23643 | T → G | Intronic | PENOC_072500 |
| contig_236 | 3287 | C → T | Non-coding region | 672bp upstream of PENOC_073480 |
| contig_342 | 7530 | T → A | Non-synonymous mutation | PENOC_088330  (298V → 298E) |
| contig_408 | 26209 | C → G | Non-coding region | 696 bp upstream of PENOC_095050 |
| contig_6 | 80023 | C → T | Synonymous mutation | PENOC_004030 |
| contig_83 | 27503 | G → T | Non-synonymous mutation | PENOC_037760  (198C → 198S) |
