# Supplementary material for: Regulatory Mechanisms of a Highly Pectinolytic Mutant of Penicillium occitanis and Functional Analysis of a Candidate Gene in the Plant Pathogen Fusarium oxysporum
Source: Front Microbiol. 2017 Sep 8;8:1627. doi: 10.3389/fmicb.2017.01627 (PMC5599776; doi:10.3389/fmicb.2017.01627)
Supplement: Supplementary Table 7 — Mycelium dry weight (mg) after 120 h growth in SM containing 0.5% of the indicated carbon source, with 170 rpm at 28°C. [file Table7.DOCX]

**Supplementary Table 7** Mycelium dry weight (mg) after 120 hours growth in SM

containing 0.5% of the indicated carbon source, with 170rpm at 28 ºC.

|  | **wild type** | **FOXG_08883Δ11** | **M22** | **M50** |
| --- | --- | --- | --- | --- |
| Sucrose | 24 | 13 | 18 | 17 |
| Cellobiose | 35 | 28 | 28 | 25 |
| Cellulose | 180 | 189 | 196 | 190 |
| CMC | 2 | 3 | 5 | 3 |
| CMC+Glucose | 15 | 11 | 21 | 24 |
